# Recognizing Leber’s Hereditary Optic Neuropathy to avoid delayed diagnosis and misdiagnosis

**DOI:** 10.3389/fneur.2024.1466275

**Published:** 2024-09-19

**Authors:** Chiara La Morgia, Maria Lucia Cascavilla, Anna Maria De Negri, Marcello Romano, Fabrizio Canalini, Silvia Rossi, Diego Centonze, Massimo Filippi

**Affiliations:** ^1^IRCCS Istituto delle Scienze Neurologiche di Bologna, Bologna, Italy; ^2^Dipartimento di Scienze Biomediche e Neuromotorie, Università di Bologna, Bologna, Italy; ^3^Department of Ophthalmology, University Vita-Salute, IRCCS Ospedale San Raffaele, Milan, Italy; ^4^Azienda Ospedaliera San Camillo-Forlanini, Rome, Italy; ^5^Azienda Ospedaliera Ospedali Riuniti Villa Sofia Cervello, Palermo, Italy; ^6^Chiesi Italia S.p.A, Parma, Italy; ^7^Department of Systems Medicine, Tor Vergata University, Rome, Italy; ^8^Unit of Neurology, IRCCS Neuromed, Pozzilli, Italy; ^9^Neurology Unit, IRCCS San Raffaele Scientific Institute, Milan, Italy; ^10^Neurorehabilitation Unit, IRCCS San Raffaele Scientific Institute, Milan, Italy; ^11^Neurophysiology Service, IRCCS San Raffaele Scientific Institute, Milan, Italy; ^12^Neuroimaging Research Unit, Division of Neuroscience, IRCCS San Raffaele Scientific Institute, Milan, Italy; ^13^Vita-Salute San Raffaele University, Milan, Italy

**Keywords:** Leber’s Hereditary Optic Neuropathy, optic nerve, visual field, Optical Coherence Tomography, retinal ganglion cell

## Abstract

Leber’s Hereditary Optic Neuropathy (LHON) is a maternally inherited optic nerve disease primarily caused by mutations in mitochondrial DNA (mtDNA). The peak of onset is typically between 15 and 30 years, but variability exists. Misdiagnosis, often as inflammatory optic neuritis, delays treatment, compounded by challenges in timely genetic diagnosis. Given the availability of a specific treatment for LHON, its early diagnosis is imperative to ensure therapeutic appropriateness. This work gives an updated guidance about LHON differential diagnosis to clinicians dealing also with multiple sclerosi and neuromyelitis optica spectrtum disorders-related optic neuritis. LHON diagnosis relies on clinical signs and paraclinical evaluations. Differential diagnosis in the acute phase primarily involves distinguishing inflammatory optic neuropathies, considering clinical clues such as ocular pain, fundus appearance and visual recovery. Imaging analysis obtained with Optical Coherence Tomography (OCT) assists clinicians in early recognition of LHON and help avoiding misdiagnosis. Genetic testing for the three most common LHON mutations is recommended initially, followed by comprehensive mtDNA sequencing if suspicion persists despite negative results. We present and discuss crucial strategies for accurate diagnosis and management of LHON cases.

## 1 Introduction

Leber’s Hereditary Optic Neuropathy (LHON) is a maternally inherited blinding disorder caused by mutations in mitochondrial DNA (mtDNA) (1). The most common mtDNA mutations associated with LHON are the 11,778/ND4 (about 75% of cases), 3,460/ND1 (about 15% of cases) and 14,484/ND6 (about 15% of cases) mutations, accounting for 90–95% of LHON cases (2). The rate of visual recovery is different for different primary mutation being higher for the 14,484/ND6 mutations (up to 70%) and much lower for the 11,778/ND4 and 3,460/ND1 mutations (about 15%) (2). Other rarer mtDNA mutations have also been linked to LHON (3). Moreover, recently, recessive forms of LHON mostly associated with *DNAJC30* gene mutations have been identified, particularly in individuals of East European ancestry (4, 5). Metabolic insult caused by mitochondrial impairment due to complex I dysfunction underlies the onset of the disease (2).

LHON prevalence is estimated approximately in 1/30.000 individuals, although it varies across countries (6). The prevalence is higher among males with a M:F ratio varying depending on the three most common mtDNA mutations. For the 14,484/ND6 mutation, the male-to-female ratio is higher reaching 8:1. Furthermore, LHON is characterized by incomplete penetrance. The probability that a carrier of the LHON mutation is affected by the disease has been previously estimated to be around 10% for females and 40–50% for males (2) even if recent studies challenged these numbers to much lower risk estimates (17.5% for males and 5.4% for females) (7–9).

Onset typically occurs in the second decade of life, but cases in childhood and late-onset have also been reported (6). The primary differential diagnosis for LHON is inflammatory optic neuritis and often the patients are misdiagnosed and treated with steroids delaying the diagnosis (10). Another important cause contributing to diagnosis delay is the difficulty in accessing a timely genetic diagnosis. A consensus conference defined the clinical diagnostic criteria and disease staging for LHON; thus, four clinical stages can be distinguished: (I) Asymptomatic; (II) Subacute (within 6 months from onset); (III) Dynamic (6–12 months from onset); (IV) Chronic (>12 months) (11).

Since 2015 idebenone has been the only approved treatment for LHON in Europe (12). Other therapeutic options such as gene therapy are currently being investigated and evaluated (13). A prompt diagnosis is crucial for initiating therapy (11). In this perspective, we will highlight the key clinical diagnostic/instrumental findings that will assist clinicians dealing with different forms of neuro-ophthalmic disorders, in early disease recognition of LHON and help avoiding misdiagnosis.

## 2 Key signs and findings/red flags useful for the correct early diagnosis of LHON

A combination of clinical signs and paraclinical examinations is necessary to reach a diagnosis of LHON. To establish the severity and the stage of the disease, the following evaluations are recommended: (1) Measurement of best-corrected visual acuity and color vision, (2) Assessment of visual fields (VF) with static or kinetic perimetry, (3) Measurement of retinal nerve fiber layer (RNFL) and ganglion cell layer (GCL) thickness with Optical Coherence Tomography (OCT) imaging.

### 2.1 Clinical signs

Typically, LHON manifests as acute or subacute central visual loss without pain during eye movements. Visual acuity rapidly deteriorates to a level worse than 20/200 reaching a nadir within approximately 6 months. Approximately 20–25% of cases experience simultaneous bilateral vision loss (14). In case of unilateral involvement, the second eye becomes affected with a median delay of 6–8 weeks. In rare cases visual loss in the second eye may occur more than a year later. Dyschromatopsia is common and typically corresponds to the degree of visual acuity loss (2, 6). Pupillary light reflexes usually remain intact due to relative sparing of melanopsin-containing retinal ganglion cells (mRGCs) that are believed to be more resistant to metabolic damage caused by mitochondrial dysfunction when compared with canonical RGCs (15, 16). However, in the subacute phase in cases with bilateral but very asymmetric involvement and in unilateral cases a relative afferent pupillary defect may occur.

In the asymptomatic stage, visual acuity is normal and only subtle color vision deficits may be observed. Fundus abnormalities such as peripapillary telangiectatic vessels and variable degrees of retinal nerve fiber layer (RNFL) increased thickness, that can vary over time, have been documented (17).

**Figure 1:**
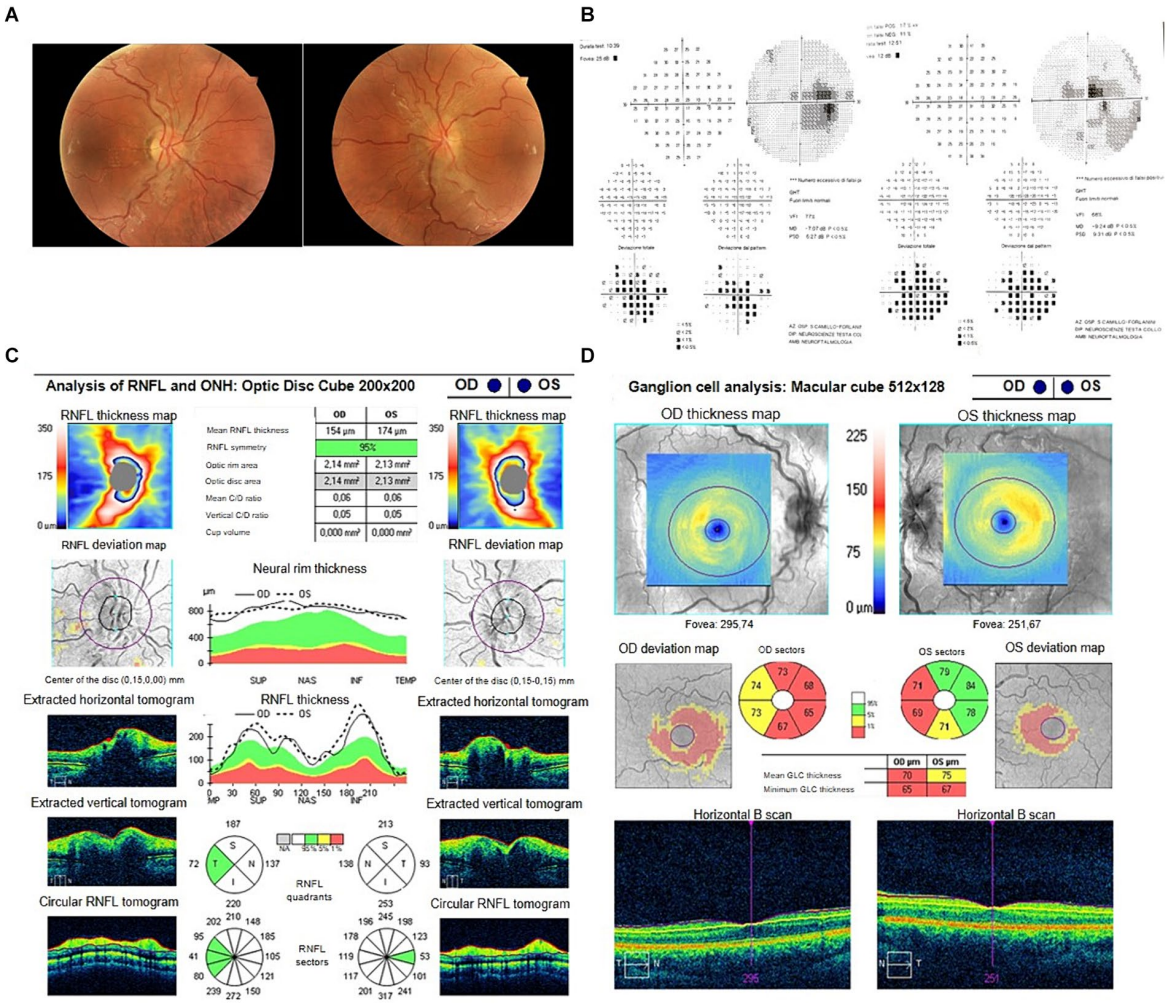
**(A)** Fundus oculi of a LHON patient in the very early stage of the disease. **(B)** Typical LHON case patient’s VF showing bilateral central scotoma. **(C)** OCT findings of a LHON patient in the early stage of the disease showing bilateral increased RNFL thickness in both eyes with initial temporal RNFL thinning in the RE. **(D)** Ganglion cell layer findings in the early stage of disease in LHON patient showing GCL defect more pronounced in the RE.

During or prior to the acute stage of vision loss, characteristic findings of LHON can be observed on fundus examination, including optic disc hyperemia, peripapillary telangiectatic blood vessels, vascular tortuosity, and RNFL swelling around the optic disc (Figure 1A). In the chronic stage, visual acuity typically remains stable, although a small percentage of patients may experience spontaneous visual recovery, whereas others may continue to present visual deterioration over time. The likelihood of visual recovery is highest with the m.14484T>C mutation (2).

### 2.2 Clinical test findings in LHON

#### 2.2.1 Visual fields

The VF testing is essential for diagnosis and monitoring visual function in various optic neuropathies, including LHON. In LHON with bilateral onset VF typically show in the subacute phase dense central or centrocecal scotomas that are unrelated to the vertical midline and preservation of the peripheral VF (Figure 1B). During the early stages, the centrocecal scotoma may present as a mild central depression (53.1% of cases) and paracentral scotoma (24.5%). The VF defect in LHON primarily affects the central 20° of vision and rarely encompasses less than the central 10°. A central scotoma (5°–20° surrounding the fixation point) generally indicates selective damage to the papillomacular bundle. Over subsequent weeks, as the disease progresses, scotoma often enlarges and becomes denser, and the VF defects worsen around the scotoma, extending from both sides or enlarging surrounding the blind spot. In the late phase, visual field defects mainly include central isopter constriction (36.7%), diffuse defects (42.9%), hemianopia or quadrantanopia (10.2%) (18).

Unlike glaucoma, where early-stage VF defects such as nasal step and arcuate scotoma are often visible, these types of defects are very rare in LHON.

After the first year following disease onset, VF can improve with the appearance of small islands of vision. These fenestrations can be beneficial for vision, especially when the density of the central scotoma simultaneously decreases.

VF defects may be rarely the only abnormality in LHON patients in the subacute phase, even in the absence of fundoscopic changes.

Static automated perimetry (SAP) or Goldmann manual kinetic perimetry have been used to assess VFs in LHON. Due to the typically poor visual acuity and potentially severe VF loss in LHON patients, Goldmann kinetic perimetry has been considered more suitable than SAP for VF testing. Goldmann manual perimetry is easier for the patient and the duration of the examination can be adjusted based on the patient’s alertness. However, manual kinetic testing shows disadvantages including the reliance on the examiner and lack of electronic storage of the results. An alternative to Goldmann manual kinetic perimetry is the more standardized semiautomated kinetic perimetry (SKP). SKP provides digitized results, uses a constant stimulus movement, and allows for measurement of the scotoma area in square degrees (deg2).

Currently, the most common VF test for LHON patients is the Humphrey VF analyzer (HVF) with a 24–2 or 30–2 Swedish interactive thresholding algorithm (SITA) strategy, using a Goldmann stimulus size III test point (Stim III). The mean deviation (MD) is a common metric to quantify the VF defect in LHON. The mean deviation represents the average age-corrected visual loss per test location weighted for the variations within and between subjects based on eccentricity. On the other hand, the MD indicates the average total deviation loss across the VF and may be problematic in case of central visual loss as the variability among test locations increases with visual impairment. In LHON, the MD often falls at the bottom end of the scale, <−30 dB. This compromises the dynamic range of the test as the patient may no longer perceive the small size III stimulus, resulting in a floor effect. Moreover, dense scotomas lead to increased fixation losses and false-negative errors, making the test “unreliable.” Consequently, the MD metric becomes insensitive to change, making it difficult to track progression or improvement in the VF deficit. In such unreliable conditions, the device fails to detect minor changes and the range between the smallest and largest values collapses, flattening the dynamic range. As a consequence, the interpretation of VF changes in patients with LHON and dense central scotomas, as well as the evaluation in clinical studies of LHON becomes challenging (19). Other strategies have been described for testing patients with low vision, such as increasing the size of the stimulus. A recent study proposes that using the Goldmann stimulus size V (Stim V) could expand the dynamic range and reduce the floor effect seen with Stim III HVF tests for LHON in the plateau stage of the disease (20).

#### 2.2.2 Optical Coherence Tomography

Optical Coherence Tomography (OCT) has been used to investigate the RNFL, optic nerve head and Ganglion Cell Layer (GCL) in unaffected carriers of LHON mutations, as well as in LHON patients in the early and atrophic stage of the diseases. Longitudinal studies on LHON patients have identified distinct RNFL changes through OCT. Fundus examination at the time of onset typically reveals pseudoedema of the retinal fibers around the optic disc, hyperemia, peripapillary telangiectasias and mild tortuosity. Asymptomatic carriers may exhibit significant thickening of the temporal RNFL compared to age-matched controls, with a slight trend towards inferior RNFL thickening in male carriers (21). This confirms the preferential involvement of the papillomacular bundle (PMB) even in subclinical LHON, which is attributed to the unfavorable energetic conditions of the small axons originating from macular parvocellular RGCs. These small fibers have a high firing rate and are highly dependent on energy resources (22). Moreover, LHON carriers show higher variability in peripapillary RNFL thickness compared to controls, as measured at different time points. RNFL variability could be explained either by a compensatory mechanism involving increased mitochondrial biogenesis or by an axonal stasis preceding RGC loss. Upon the onset of symptoms, the thickening of the temporal and inferior RNFL is followed by thickening of the superior and nasal sectors, while the temporal sector starts thinning at an early stage (23) (Figure 1C). These changes correspond to the appearance of disc edema, hyperemia and microvascular alterations observed during fundus examination (24). The RNFL thickening seems primarily related to axonal swelling, which has been proposed to be a sign of impaired axonal transport, with redistribution of mitochondria within the dysfunctional RGCs. This particularly affects the prelaminar, unmyelinated portion of the axons at the optic nerve head (2). The subacute phase of LHON has been proven to be more variable and progressive than previously understood, characterized by a series of events lasting at least 3 months (23). Around 3 months after onset, temporal thinning becomes noticeable, followed by evident thinning of the superior and inferior regions by 9 months due to early atrophy. After 6 months from onset, diffuse optic atrophy becomes evident. At this stage patients present a significant reduction in visual acuity, which usually reaches its lowest point within 6 months from onset. In the atrophic stage of the disease, patients with visual recovery maintain a thicker RNFL than the those without visual recovery in all quadrants except for the temporal quadrant.

OCT also allows to investigate the size of the optic nerve head (ONH). LHON carriers show a larger ONH compared to both symptomatic patients and healthy controls. This larger ONH size has been suggested to likely be associated with less crowding of RGC axons, representing a favorable prognostic factor that may protect LHON carriers from developing the acute phase of the disease (25). In the chronic stage a diffuse optic nerve pallor becomes evident, corresponding to diffuse optic atrophy, as assessed by OCT. A larger ONH may also influence visual recovery since affected LHON with visual recovery also had a significantly larger ONH. Moreover, LHON patients carrying the 14,484/ND6 mutation had larger optic discs compared to those with other mutations and, in fact, this is the mutation associated with a more favorable prognosis (25).

OCT analysis allows also the segmentation of the retinal layers and in particular of the GCL. In LHON patients the macular RGC layer shows diffuse thinning and the involvement of RGCs has been demonstrated in the very acute stage of the disease. In fact, ganglion cell analysis can detect early damage to the macular RGCs, which precedes axonal loss and thinning of the RNFL and even the appearance of visual symptoms (26) (Figure 1D). Topographical analysis of the macular region demonstrated an early and extended involvement of the inner ring, compared to the outer ring and progresses in a centrifugal and spiral pattern resembling the anatomic distribution of the papillomacular bundle fibres (26). Additionally, in terms of total macular thickness, the nasal quadrant of the outer ring tends to become thinner earlier (within 3–6 months) than the superior and inferior quadrants (within 6–9 months). These findings overall confirm the capability of OCT in evaluating macular structure and the early involvement of the nasal sectors of the macular region corresponding to the PMB in LHON.

#### 2.2.3 Optical Coherence Tomography angiography

OCT angiography (OCT-A) is a non-invasive imaging technique that provides high-resolution images of retinal and peripapillary capillaries by visualizing vascular flow via motion contrast (27). This innovative technique offers several advantages over fluorescein angiography, which has been the gold standard to evaluate retinal circulation in LHON patients (28–30). In addition to superior resolution, OCT-A allows for the visualization of vascular structures in an arbitrarily selected depth of retinal layers without the need to administer a contrast agent (31, 32). OCT-A enables the evaluation of optic nerve head vessel density (VD) and radial peripapillary capillaries (RPC). By using the split-spectrum amplitude-decorrelation angiography algorithm, vascular perfusion can be quantified. Studies have identified significant peripapillary microvascular changes throughout the different stages of LHON progression (33–35). The microvascular changes in the temporal sector, implicating the PMB, occur prior to thinning of the RNFL and mirror the changes in the GC-IPL (33). This supports an active role of the retinal microvasculature in the process of disease during LHON conversion, leading to an irreversible wave of axonal injury (RNFL thickening) and subsequent irreversible loss of RGC (GC-IPL thinning). No statistically significant differences of VD were found between LHON-unaffected individuals and controls in any sector. However, in the early stage of LHON, the VD were significantly reduced in the temporal sector and in the temporal and inferotemporal sectors compared to both LHON-unaffected individuals and controls, respectively. In LHON-late subacute stage VD were significantly reduced in whole, temporal, superotemporal and inferotemporal sectors compared with LHON-unaffected and controls. In LHON-chronic stage, the VD was reduced in all sectors when compared to all the other stages, including the control group. At late chronic stage a generalized thinning affects retinal microvasculature, RNFL and GC-IPL (33).

#### 2.2.4 Brain and orbit magnetic resonance imaging findings

In acute classical LHON, brain MRI is usually normal and there is no evidence of demyelinating lesions in the white matter of the brain, which distinguishes from optic neuritis associated with demyelinating diseases (36). However, in some cases, LHON patients in the acute phase can show hyperintensity in the optic nerve/optic chiasm and rarely, enhancement of the optic nerve/optic chiasm has been observed (37, 38). Cases of LHON resembling neuromyelitis optica have been also reported (39–41). Moreover, the co-occurrence of LHON and MS, the so-called Harding’s disease, has also been reported (42) and in LHON patients without MS white matter changes can be frequently observed (43). Furthermore, the use of MRI is helpful also for differentiating LHON from compressive and/or infiltrative processes of the optic nerves.

## 3 The importance of family history and environmental factors

Since LHON is a maternally inherited disorder it is crucial to investigate for the possible presence of other optic neuropathy cases along the maternal side. In fact, LHON mutations affect the mtDNA, which is inherited exclusively through the maternal line. An appropriate genetic counselling is needed not only for accurate diagnosis but also for providing guidance to the individuals connected through the maternal lineage, including the risk of transmission of mtDNA mutation to siblings. In fact, the large majority of LHON mutations are homoplasmic, meaning that all the individuals along the maternal lineage are at risk of carrying and transmitting the LHON mutation to siblings. Moreover, since a preventive therapy for LHON carriers is not yet available, it is highly recommended to provide comprehensive instructions regarding potential triggers of the disease, such as smoking and alcohol consumption and also about the potential toxicity of some drugs (44).

## 4 Differential diagnosis of LHON

In the acute phase, the main differential diagnosis for LHON is represented by inflammatory optic neuropathies, which include retrobulbar optic neuritis and anterior papillitis (45). The main clinical clues for differential diagnosis with inflammatory optic neuritis are the presence of ocular pain at eye movements which occurs in 90% of cases, the occurrence of visual recovery, spontaneously or after steroid therapy within 1 month from onset, the peculiar and specific funduscopic and OCT features of LHON and the evidence of optic nerve hyperintensity with gadolinium enhancement at brain and orbit MRI. Fluorescein angiography in the large majority of cases shows the absence of leakage at the optic nerve head in LHON, aiding in the differentiation of LHON from inflammatory papillitis and other optic neuropathies (46). In general, in the case of unilateral and especially rapidly sequentially bilateral optic neuropathy in a young male without pain or spontaneous visual recovery the suspicion of LHON should be always raised, also in the absence of a clear family history. In this context another important differential for LHON is represented by Neuromyelitis Optica Spectrum Disorder-optic neuritis and MOG antibody-associated disease (MOGAD)-optic neuritis in which the more frequent bilateral involvement, and the usual more severe visual outcome and severity of optic atrophy make the differential diagnosis more difficult than for the classical Multiple Sclerosis-related optic neuritis (45).

Other relevant differential diagnosis for LHON are represented by toxic-nutritional optic neuropathies which are typically characterized by subacute and painless central visual loss with central scotoma and temporal pallor. The clinical course (i.e., recovery after stopping toxins and/or supplementing vitamins) is of help in differential diagnosis (47).

## 5 Genetic testing

In case of clinical suspicion of LHON, it is mandatory to undergo genetic testing for the three most common LHON mutations (11,778/ND4, 3,460/ND1 and 14,484/ND6) which account for 90–95% of LHON. In cases where the initial testing is negative but the clinical suspicion remains high, a comprehensive sequencing of the entire mtDNA should be performed. If mtDNA is completely negative, and there is evidence of possible recessive inheritance, exome sequencing should also be performed including recessive LHON in-silico gene panel screening (including genes associated to recessive LHON and in particular *DNAJC30* gene).

## 6 Final considerations

**Figure 2:**
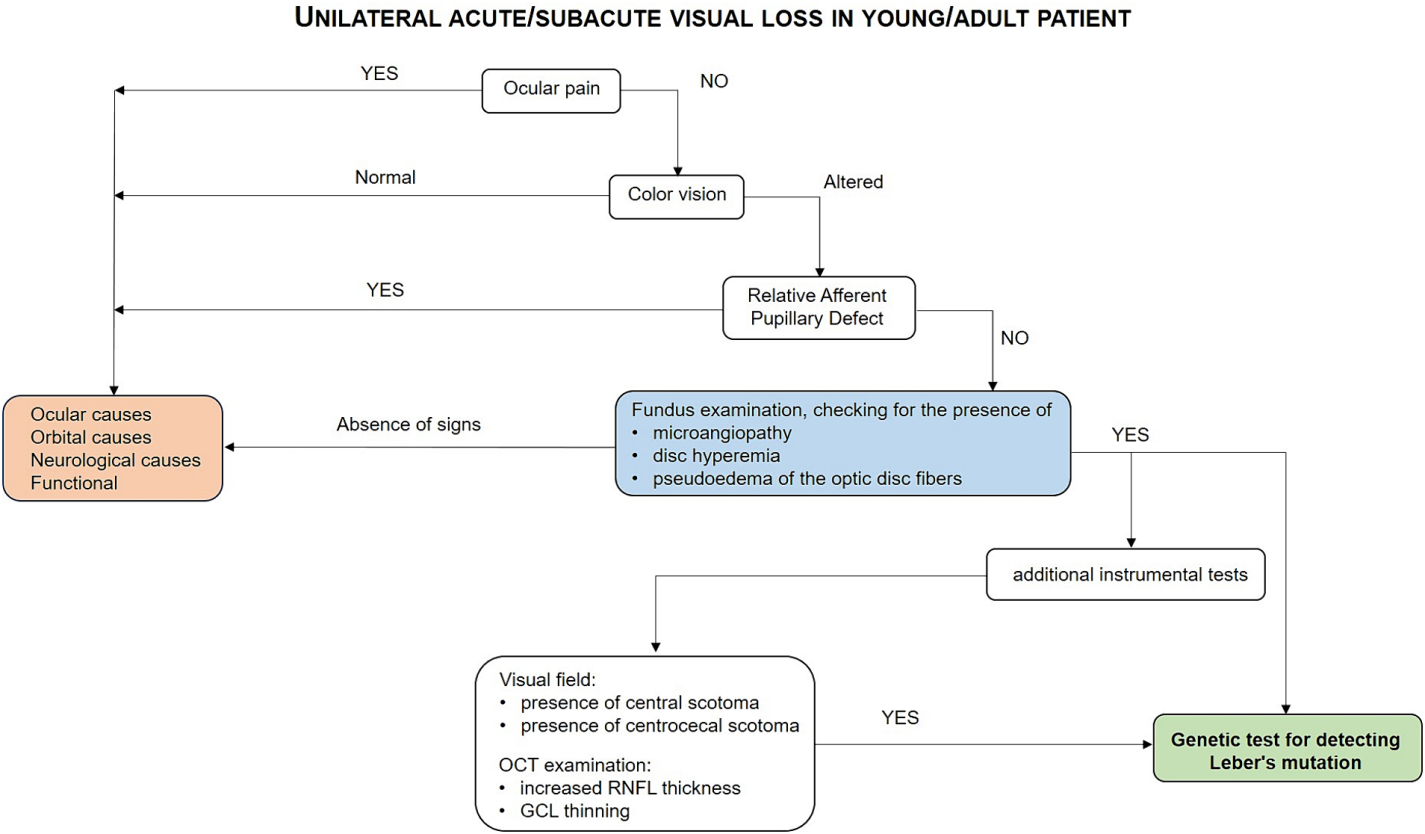
Flow-chart guiding the diagnostic process for LHON.

LHON is a maternally-inherited optic nerve disease in most instances, which is characterized by painless acute loss of visual acuity due to mitochondrial dysfunction affecting RGCs leading in the majority of cases to permanent visual loss. The typical natural history of the disease and the use of clinical tests including visual fields and particularly OCT helps in reaching the diagnosis in the very early stage of the disease avoiding misdiagnosis and inappropriate diagnostic exams and therapies. The early diagnosis is instrumental for starting the appropriate therapy (see diagnostic flow-chart in Figure 2) and to avoid misdiagnosis and inappropriate treatments. The only approved therapy for LHON at the moment is idebenone. The results of the open-label natural history-controlled trial on LHON have been recently published confirming idebenone long-term efficacy in both subacute and chronic LHON patients (48). Moreover, gene therapy trials on LHON patients carrying the 11,778/ND4 mutation have been conducted demonstrating a clinically relevant and sustained improvement in visual acuity compared to natural history data (49).

## Data Availability

The original contributions presented in the study are included in the article/supplementary material, further inquiries can be directed to the corresponding author.
